# Impact of Global Normalization in fMRI Acupuncture Studies

**DOI:** 10.1155/2012/467061

**Published:** 2012-12-17

**Authors:** Jinbo Sun, Wei Qin, Lingmin Jin, Minghao Dong, Xuejuan Yang, Yuanqiang Zhu, Yang Yang, Karen M. von Deneen, Qiyong Gong, Jie Tian

**Affiliations:** ^1^Life Sciences Research Center, School of Life Sciences and Technology, Xidian University, Xi'an, Shaanxi 710071, China; ^2^Huaxi MR Research Center (HMRRC), Department of Radiology, West China Hospital, Sichuan University, Sichuan 610041, China; ^3^Institute of Automation, Chinese Academy of Sciences, Beijing 100190, China

## Abstract

Global normalization is often used as a preprocessing step for dispelling the “nuisance effects.” However, it has been shown in cognitive and emotion tasks that this preprocessing step might greatly distort statistical results when the orthogonality assumption of global normalization is violated. The present study examines this issue in fMRI acupuncture studies. Thirty healthy subjects were recruited to evaluate the impacts of the global normalization on the BOLD responses evoked by acupuncture stimulation during *De-qi* sensation and tactile stimulation during nonpainful sensations. To this end, we compared results by conducting global normalization (PSGS) and not conducting global normalization (NO PSGS) based on a proportional scaling model. The orthogonality assumption of global normalization was violated, and significant changes between BOLD responses for NO PSGS and PSGS were shown in most subjects. Extensive deactivations of acupuncture in fMRI were the non-specifically pernicious consequences of global normalization. The central responses of acupuncture during *De-qi* are non-specifically activation-dominant at the somatosensory-related brain network, whose statistical power is specifically enhanced by PSGS. In conclusion, PSGS should be unjustified for acupuncture studies in fMRI. The differences including the global normalization or not may partly contribute to conflicting results and interpretations in previous fMRI acupuncture studies.

## 1. Introduction

Blood oxygen level-dependent (BOLD) functional magnetic resonance imaging (fMRI) is increasingly used to investigate central mechanism of acupuncture [1–7]. However, few hypotheses have been recognized without debate in fMRI acupuncture studies. Various kinds of differences could bring about the disparate results in fMRI acupuncture studies [8], particularly the differences in data processing and statistical analysis methods [8–11]. As a questionable data processing step which is at risk of changing the interpretation of the statistical results both qualitatively (changing the sign of the results) and quantitatively [12–14], the normalization of the global signal was adopted in many acupuncture fMRI studies [3, 5, 7, 15–21]. This paper attempts to evaluate the validity and impact of the normalization of the global signal in acupuncture fMRI studies.

It is known that global normalization was usually employed to dispel global effects, which were considered as “nuisance effects” [12, 13, 22]. Its employment was restricted to the assumption that global effects are orthogonal to the variation of the task-induced effect [12, 13, 22]. In this case, the global signal (estimator of the global effects which is defined by the simple average of all voxels within the brain at each time point) was usually normalized in a proportional scaling or ANCOVA model [12–14, 22, 23]. When the task-induced effect is strong enough to contaminate the global signal and to break the orthogonality assumption, the global normalization would be unjustified, often reduce activations, and introduce artificial deactivations [13, 14, 22, 24, 25]. The impact of global normalization in fMRI studies in the domain of emotion research [24], other cognitive tasks [12, 13], and on resting state correlations [25] has been evaluated. So far, however, none of the acupuncture studies in fMRI took the impacts of global normalization into account. Therefore, it raised questions on whether the orthogonality assumption of global normalization is satisfied in acupuncture fMRI studies or not. Furthermore, if violated, the impacts of global normalization on activations and deactivations need to be determined in acupuncture fMRI studies. 

The current study set out to (1) assess the validity of using proportional scaling to normalize the global signal in acupuncture fMRI studies and (2) examine the impacts of global normalization on activations and deactivations in acupuncture fMRI studies. To this end, we compared results of the same dataset obtained during acupuncture stimulation by conducting and not conducting global normalization based on a proportional scaling model. Besides acupuncture, a superficial tactile stimulus (noninvasive) was employed to explore the acupuncture-specific results.

## 2. Materials and Methods

### 2.1. Subjects

Participants were recruited from a group of 30 college students (15 males and 15 females; ages 22.2 ± 1.3 years). All subjects were right-handed with normal or corrected-to-normal vision. Subjects were acupuncture-naïve, had no history of major medical illnesses, head trauma, neuropsychiatric disorders, or any prescription medications one month preceding the experiment, and did not have any contraindications to exposure to a high magnetic field. All subjects gave written and informed consent after the experimental procedures were fully explained. All research procedures were approved by the West China Hospital Subcommittee on Human Studies and were conducted in accordance with the Declaration of Helsinki.

### 2.2. Experimental Procedures

For each subject, functional scans of tactile stimulation were collected prior to anatomical scans, and functional scans of acupuncture stimulation were taken after the anatomical scans. The intervals between runs were 5–10 minutes. The order of tactile stimulation and acupuncture stimulation was not given to the subjects. During fMRI scanning, all subjects were instructed to keep their eyes closed to prevent them from actually observing the procedures. Subjects were told that acupuncture was to be performed with different techniques that would generate different sensations during needling. Because the subjects were acupuncture naïve, they would be unable to discriminate the tactile stimulation control from real acupuncture until they experienced real acupuncture.

**Figure 1:**
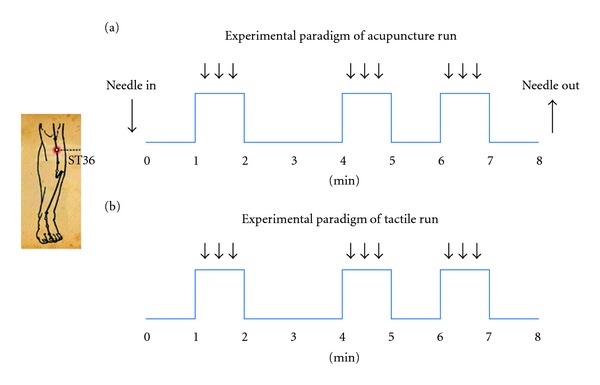
Experimental paradigm: scans of acupuncture stimulation (a) and tactile stimulation (b) lasted 8 min and consisted of three one-minute acupuncture/tactile stimulations.

Acupuncture stimulation was performed at acupoint ST36 on the right leg (Zusanli, located in the tibialis anterior muscle four fingerbreadths below the lower margin of the patella and one fingerbreadth lateral from the anterior crest of the tibia). The fMRI paradigm for the acupuncture stimulation run lasted for 8 minutes and consisted of three one-minute acupuncture manipulations (Figure 1(a)). The needle was inserted perpendicularly to a depth of 2-3 cm before the scan started. A one-minute baseline period was held preceding the first acupuncture stimulation. The interval between the first two acupuncture manipulations was two minutes, while the second and third acupuncture manipulations were separated by an interval of one minute. Scanning was then continued for another minute after the third manipulation. During the acupuncture procedure, the needle was rotated manually clockwise and counterclockwise for one minute at a rate of 60 times per minute. The stimulation was administered by a balanced “tonifying and reducing” technique using a sterile disposable 38 gauge stainless steel acupuncture needle (0.3 mm × 40 mm). After the scan ended, the needle was extracted. The tactile stimulation run was performed at acupoint ST36 on the right leg with a size 5.88 Von Frey monofilament. The paradigm of tactile stimulation was matched to that of the acupuncture stimulation run (Figure 1(b)). During the tactile manipulations, the monofilament tapped the skin gently at a rate of 60 times per minute. The acupuncture and tactile procedures were conducted by the same experienced and licensed acupuncturist.

In the end, the subjects were facilitated by the acupuncturist to quantify their sensations using a 10-point visual analogue scale (VAS) to rate their *De-qi* experience felt during the acupuncture run [11, 26, 27]. To quantitatively summarize the full multivariate breadth and depth of the *De-qi* sensations for each subject, the VAS index was calculated [11, 26, 27]. Because sharp pain was considered an inadvertent noxious stimulation and evoked different BOLD responses to the *De-qi* sensation [3], we excluded subjects from further analysis if they experienced sharp pain. Among the 30 participants, two reported sharp pain during both runs, three experienced sharp pain only in the acupuncture run, and one felt sharp pain only in the tactile run. Thus, 25 subjects in the acupuncture run and 27 subjects in the tactile run remained for data analysis.

### 2.3. fMRI Scanning Procedure

Imaging data were collected from a 3T Siemens scanner (Allegra; Siemens Medical System) at the Huaxi MR Research Center, West China Hospital of Sichuan University, Chengdu, China. A standard birdcage head coil was used, along with restraining foam pads to minimize head motion and to diminish scanner noise. Thirty axial slices (FOV = 240 mm × 240 mm, matrix = 64 × 64, thickness = 5 mm) parallel to the AC-PC plane covering the whole brain were obtained using a T2*-weighted single-shot, gradient-recalled echo planar imaging (EPI) sequence (TR = 2,000 ms, TE = 30 ms, and flip angle = 90°). The scan covered the entire brain including the cerebellum and brainstem. After the functional run, high-resolution structural information on each subject was acquired using 3D MRI sequences with a voxel size of 1 mm^3^ for anatomical localization (TR = 2.7 s, TE = 3.39 ms, matrix = 256 × 256, FOV = 256 mm × 256 mm, flip angle = 7°, in-plane resolution = 1 mm × 1 mm, slice thickness = 1 mm).

### 2.4. fMRI Data Analysis

Preprocessing and statistical analyses at both the individual level and group level were performed using the Statistical Parametric Mapping software (SPM5, http://www.fil.ion.ucl.ac.uk/spm/). Initially, the first 5 time points were discarded in order to avoid the instability of the initial MRI signal. The remaining images were realigned to the first volume. Three subjects in the acupuncture run and four subjects in the tactile run exceeded our rigorous motion threshold of less than 1 mm spatial displacement in any direction. Ultimately, 22 subjects (10 males) in the acupuncture run and 23 subjects (12 males) in the tactile run remained. Subsequently, the images were normalized to the standard EPI template and resampled to a voxel size of 3 × 3 × 3 mm^3^ and then smoothed spatially using a 6 mm full-width-at-half maximum (FWHM) isotropic Gaussian kernel to decrease spatial noise. The global normalization by proportional scaling was applied for a single processing flow (PSGS) and was not applied to another processing flow (NO PSGS). Then, the time series from each voxel were high-pass filtered (1/235-Hz cutoff) to remove low-frequency noise and signal drift. For each subject, the preprocessed fMRI data were then submitted for fixed-effects model analyses using the general linear model (GLM) performed at each voxel across the whole brain. After acquiring the contrast images, individual level analyses were accomplished, and statistical parametric maps for the *t* statistics (spmT) were then generated for each contrast image. At the group level, the random-effects model analysis was performed based on inference images (i.e., *t*-test for contrast images) from the individual level analysis. 

In order to examine whether the orthogonality assumption was satisfied or not, the correlation coefficients (*R*-value) of the global signal and the reference vector (the experimental paradigm convolved the hemodynamic response function) (viz., “GS-RV *R*-value”) were calculated for each subject. Besides, the averaged *R*-value of the reference vector and time series of the BOLD signal across the whole brain voxels (viz., “AVER-WB-RV *R*-value”) were calculated for each subject for NO PSGS and PSGS, respectively. The changes in the “AVER-WB-RV *R*-value” (PSGS–NO PSGS) were also acquired. Then, the correlations of the “GS-RV *R*-value” and “AVER-WB-RV *R*-value” of NO PSGS, as well as the correlations of the “GS-RV *R*-value” and the changes of the “AVER-WB-RV *R*-value” were calculated. 

To evaluate the impact of global normalization, firstly, the number of activations and deactivations for each subject and for the group results was calculated for PSGS and NO PSGS, respectively. At the individual level, two thresholds were selected, a conservative one: *P* < 0.00001, uncorrected and a liberal one: *P* < 0.01, uncorrected. At the group level, three thresholds were selected, a conservative one: *P* < 0.00001, uncorrected, a moderate one: *P* < 0.001, uncorrected, and a liberal one: *P* < 0.01, uncorrected. The percentages of the changes in the activations and deactivations numbers ((PSGS-NO PSGS)/NO PSGS*100%) for each subject and for the group results were calculated, respectively. The ratios of activation divided by deactivation for each subject and for the group results were also compared between NO PSGS and PSGS. The histogram of the whole brain's *T*-values for the group results was plotted for PSGS and NO PSGS, respectively. Secondly, a paired *t*-test of the BOLD responses was carried out based on the contrast file of each subject between PSGS and NO PSGS. Two thresholds were selected, a moderate one: *P* < 0.001, uncorrected, and a liberal one: *P* < 0.01, uncorrected. The histogram of the whole brain's *T*-values of the paired *t*-test results was also plotted for each group. Thirdly, the conjunction analyses were implemented based on group results map of the one-sample *t*-test for PSGS and NO PSGS. A conservative threshold: *P* < 0.00001, uncorrected, a moderate threshold: *P* < 0.001, uncorrected, and a liberal threshold: *P* < 0.01, uncorrected were selected. Four categories were defined for activations and deactivations, respectively, namely, “stronger,” “weaker,” “disappeared” and “arisen.” The “stronger” category was the regions which were significant for both NO PSGS and PSGS, and their *t*-values were larger for PSGS than NO PSGS. The “weaker” category was the regions which were significant for both NO PSGS and PSGS and their *t*-values were smaller for PSGS than NO PSGS. The “disappeared” category was the regions which were significant for NO PSGS but were nonsignificant for PSGS. The “arisen” category was the regions which were nonsignificant for NO PSGS but were significant for PSGS. Finally, the amplitude of BOLD signal changes (ABSC) for PSGS and NO PSGS was calculated, respectively, masked by the “stronger” and, “weaker” regions of activations in the conjunction analyses maps.

For exploring the authentic acupuncture-induced BOLD response, the group results of the one sample *t*-test in the acupuncture run for NO PSGS were mapped and listed. The specific BOLD response evoked by acupuncture stimulation was explored based on a two-sample *t*-test between the acupuncture run and tactile run for NO PSGS.

## 3. Results

### 3.1. Correlations among the Reference Vector, Global Signal, and Averaged *R*-Value of the Whole Brain (Figures 2(a) and 2(b) and Figures S1 and S2)

**Figure 2:**
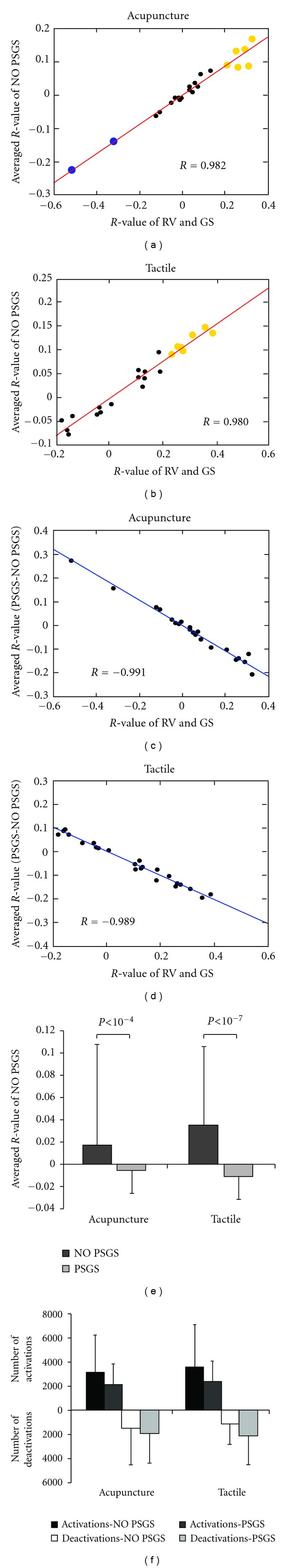
Correlations between the global signal (GS), reference vector (RV), and voxel-level time series. (a) and (b) indicate the correlation between the GS-RV *R*-value and AVER-WB-RV *R*-value (NO PSGS) for the acupuncture and tactile runs, respectively. Dots in each panel represent the subjects. The larger dots in orange and blue indicate the significant values of positive and negative correlations, respectively (*P* < 0.05, corrected). (c) and (d) manifest the linear relationship between the GS-RV *R*-value and changes in the AVER-WB-RV *R*-value (PSGS-NO PSGS) for the acupuncture and tactile runs, respectively. Dots also represent the subjects. (e) shows the mean and standard deviation (SD) of the AVER-WB-RV *R*-value across subjects for NO PSGS with a dark grey bar and for PSGS with a light grey bar in the acupuncture and tactile runs, respectively. (f) shows the mean and SD of the number of activations and deactivations across subjects for NO PSGS and PSGS. The numbers of activations for NO PSGS and PSGS are shown in black and dark grey bars, respectively. The numbers of deactivations for NO PSGS and PSGS are shown in white and light grey bars, respectively. Each number is calculated at *P* < 0.00001, uncorrected.

For acupuncture stimulation, the “GS-RV *R*-values” were significant for eight subjects (*P* < 0.05, corrected, 6 positive correlations, and 2 negative correlations in Figure 2(a)). For tactile stimulation, seven subjects' “GS-RV *R*-values” were significant (*P* < 0.05, corrected, all positive correlations in Figure 2(b)). Figures S1 and S2 (see Supplementary Material available online at doi:10.1155/2012/467061) showed the “GS-RV *R*-value” of each subject and plotted the RV and GS of representative subjects. The correlations of the “GS-RV *R*-value” and the “AVER-WB-RV *R*-value” (NO PSGS) are shown in Figures 2(a) and 2(b). Most linear relations were between these two groups of values for acupuncture (*R* = 0.982) and tactile stimulation (*R* = 0.980), indicating that the “GS-RV *R*-value” which averaged the time series and the “AVER-WB-RV *R*-value” which averaged the correlation coefficient were consistent.

### 3.2. Impact of PSGS at the Individual Level (Figures 2(c)–2(f), Table 2)

The correlation of the “GS-RV *R*-value” and the changes in the “AVER-WB-RV *R*-value” (PSGS-NO PSGS) are shown in Figures 2(c) and 2(d). Most linear relations (inverse correlation) were between these two groups of values for acupuncture (*R* = −0.991) and tactile stimulation (*R* = −0.989), indicating that the higher the correlation between GS and RV, the greater the changes (decrease) in the BOLD responses produced by the normalization of global signal. The mean and standard deviation (SD) of the AVER-WB-RV *R*-value across subjects for NO PSGS and PSGS are shown in Figure 2(e). The definite values and the differences between NO PSGS and PSGS are listed in Table S1. The mean values ranged from 0.018 to −0.005 for acupuncture and from 0.036 to −0.011 for tactile stimulation, which were both significantly changed (*p* < 10^−4^ for acupuncture and *p* < 10^−7^ for tactile stimulation). 

Figure 2(f) shows the mean and SD of the number of activations and deactivations (*P* < 0.00001, uncorrected) across subjects for NO PSGS and PSGS. The definite values and the ratio of changes between NO PSGS and PSGS are listed in Table 1. The activations of most subjects were reduced, while the deactivations were increased. The mean ratios of changes of the activations were −33% for both acupuncture and tactile stimulations. The deactivation increased 25% on average for acupuncture and 83% on average for tactile stimulation. Very similar results are manifested in Table S2 which show the number of activations and deactivations at *P* < 0.01, uncorrected for NO PSGS and PSGS. The ratios of activation divided by deactivation for NO PSGS and PSGS are listed in Table S3. For NO PSGS, the ratio was 194% or 174% for acupuncture and 297% or 189% for tactile stimulation at *P* < 0.00001, uncorrected or *P* < 0.01, uncorrected, indicating activations were greater in number than deactivations for both stimulations. However, for PSGS, the number of activations was equivalent to and even less than the number of deactivations in both stimulations (the ratio was 105% for acupuncture and 109% for tactile stimulation at *P* < 0.00001, uncorrected, while it was 88% for acupuncture and 78% for tactile stimulation at *P* < 0.01, uncorrected).

### 3.3. Impact of PSGS at the Group Level (Figure 3, Table 1, and Table S3)

**Figure 3:**
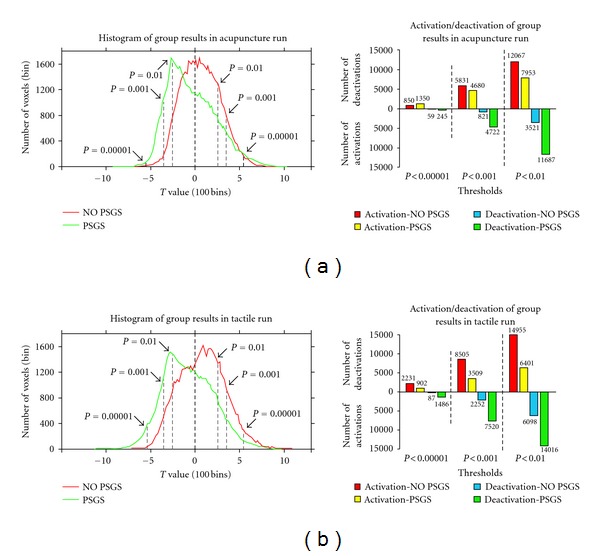
Histogram and activation/deactivation of the group results for NO PSGS and PSGS. The left part of (a) and (b) indicates the histogram of the group results based on the random effects model (REM) in acupuncture and tactile runs respectively. The dark grey line shows the histogram for NO PSGS and the light grey line shows the one for PSGS. Three thresholds were marked on the histogram, *P* < 0.00001, uncorrected, *P* < 0.001, uncorrected, and *P* < 0.01, uncorrected. The number of activations and deactivations of the group results at the three thresholds for NO PSGS and PSGS is shown in the right part of (a) and (b).

Histograms of the one sample *t*-test group results for NO PSGS and PSGS are shown in Figure 3. The right part of Figure 3, Table 1, and Table S2 shows the number of group-level activations and deactivations and their ratios of changes for NO PSGS and PSGS. For NO PSGS, the *t* distribution of acupuncture was bell-shaped with a mean greater than zero. The *t* distribution of tactile stimulation for NO PSGS was positively skewed. Group-level deactivations at *P* < 0.00001, uncorrected, were few in both stimulations for NO PSGS (59 and 87 voxels). However, for PSGS, the *t* distribution for both acupuncture and tactile stimulations was negatively skewed with the peak at about *t* = 2.5 (*P* < 0.01, uncorrected). Deactivations consistently increased at *P* < 0.00001, uncorrected, *P* < 0.001, uncorrected and *P* < 0.01, uncorrected, compared to NO PSGS for both stimulations, increasing 315%, 475%, and 232% for acupuncture and 1608%, 234%, and 130% for tactile stimulation. Activations were reduced by 20% and 34% for acupuncture and 59% and 57% for tactile stimulation at *P* < 0.001, uncorrected, and *P* < 0.01, uncorrected. Interestingly, the variation trends for the number of activations at *P* < 0.00001, uncorrected, were different between stimulations, that is, significantly increased (+59%) in acupuncture and significantly reduced (−60%) in tactile stimulation for PSGS. The ratios of activation divided by deactivation ranged from 1441%, 710%, and 598% for NO PSGS to 551%, 99%, and 68% for PSGS in acupuncture and from 2564%, 378%, and 245% for NO PSGS to 61%, 47%, and 46% for PSGS in tactile stimulation.

### 3.4. Paired *t*-Test between NO PSGS versus PSGS (Figure 4, Table 2)

**Figure 4:**
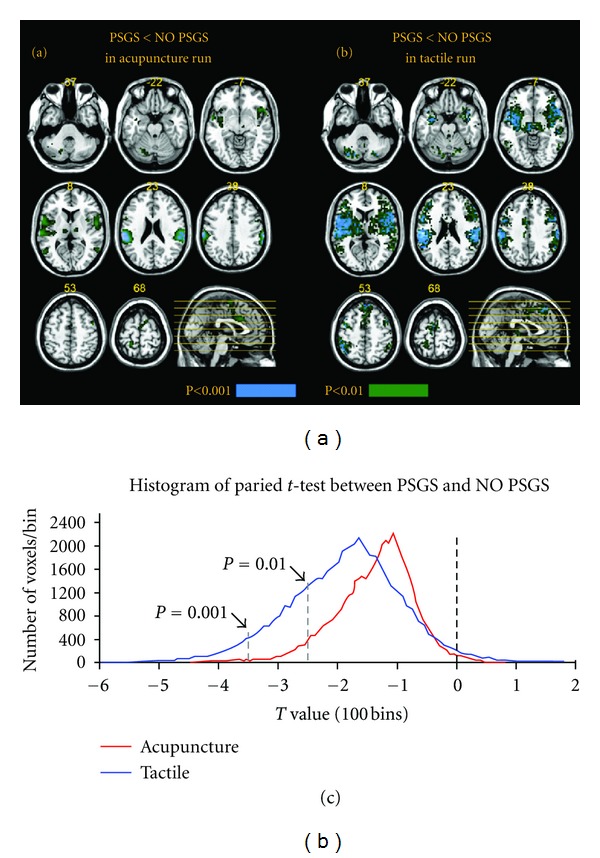
Maps and histogram for the paired *t*-test between PSGS and NO PSGS. (a) and (b) indicate the paired *t*-test map for PSGS < NO PSGS in the acupuncture and tactile runs, respectively. Two thresholds were selected and overlapped on the map: *P* < 0.001, uncorrected (colored in blue), and *P* < 0.01, uncorrected (colored in green) with 5 contiguous voxels, respectively. (c) shows the histogram of the paired *t*-test between PSGS and NO PSGS. The red line represents the acupuncture run, and the blue line indicates the tactile run. *P* < 0.001, uncorrected, and *P* < 0.01, uncorrected, are marked on the histogram.

Figure 4(c) shows the histogram of the paired *t*-test between PSGS and NO PSGS. Most *t*-values were less than zero in both stimulations, indicating the individual-level's *t*-values of most voxels for PSGS were less than those for NO PSGS. The peak value of the *t* distribution was less for tactile stimulation than for acupuncture. More voxels were shown in tactile stimulation at *P* < 0.001, uncorrected, or *P* < 0.01, uncorrected. Significant brain regions of the paired *t*-test between PSGS and NO PSGS are mapped in Figures 4(a) and 4(b). For acupuncture, the BOLD responses of the ipsilateral inferior frontal gyrus, bilateral primary somatosensory cortex (SI), bilateral inferior parietal lobule (IPL), contralateral postcentral gyrus, contralateral insula, and contralateral superior temporal gyrus were significantly reduced in PSGS. For tactile stimulation, more regions' BOLD responses were significantly reduced for PSGS, including the bilateral inferior frontal gyrus, bilateral middle frontal gyrus, bilateral precentral gyrus, SI, postcentral gyrus, bilateral IPL, bilateral thalamus, bilateral insula, bilateral claustrum, bilateral superior temporal gyrus, bilateral transverse temporal gyrus, and some regions in the cerebellum. Detailed results including coordinates, maximum *t*-values and sizes are summarized in Table 2.

### 3.5. Changes of One-Sample-Based Group Results between NO PSGS and PSGS (Figures 5 and 6, Figure S3, and Tables 3 and 4)

**Figure 5:**
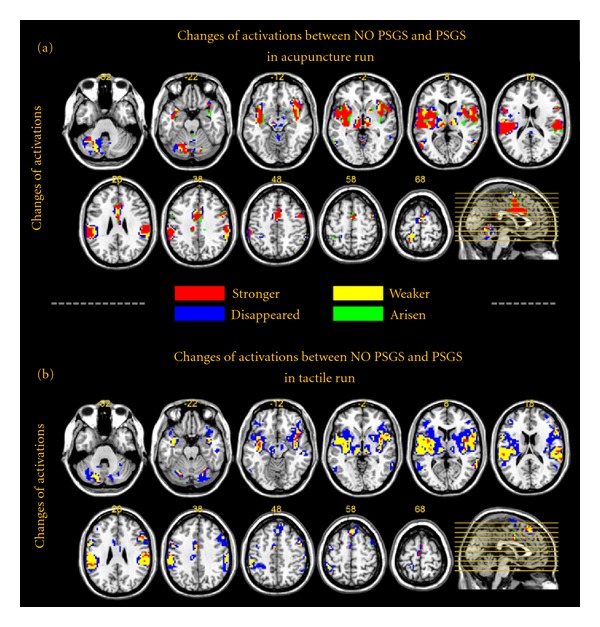
Changes in activations between NO PSGS and PSGS. The map is based on REM group results at *P* < 0.001, uncorrected with 5 contiguous voxels. (a) and (b) show the changes in activations in the acupuncture and tactile runs, respectively. For each panel, the “stronger” regions are colored in red, delegating the extent of the activations that are significant for both NO PSGS and PSGS, and show a larger absolute value of the *t*-value for NO PSGS. The “weaker” regions are colored in yellow, delegating the extent of the activations that are significant for both NO PSGS and PSGS, and show a smaller absolute value of the *t*-value for NO PSGS. The “disappeared” regions are colored in blue, delegating the extent of the activations that are significant for NO PSGS but nonsignificant for PSGS. The “arisen” regions are colored in green, delegating the extent of the activations that are nonsignificant for NO PSGS, but are significant for PSGS.

**Figure 6:**
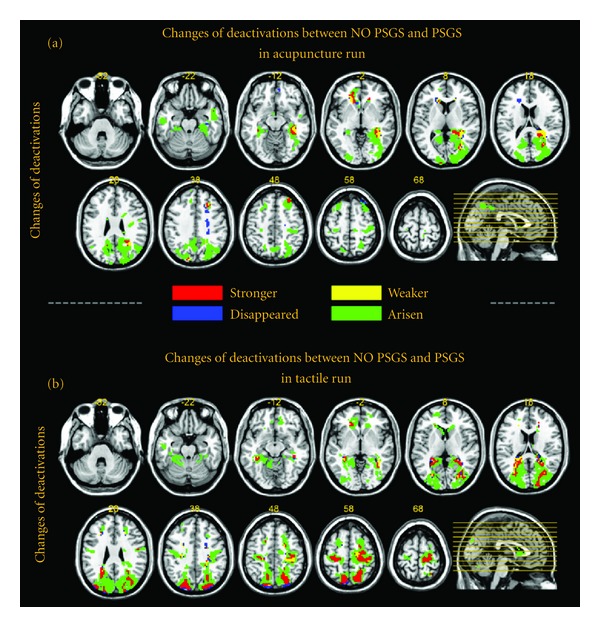
Changes in deactivations between NO PSGS and PSGS. The map is based on REM group results at *P* < 0.001, uncorrected with 5 contiguous voxels. (a) and (b) show the changes in activations in the acupuncture and tactile runs, respectively. For each panel, the meaning for each color is identical to that in Figure 5.

The changes in activations and deactivations between PSGS and NO PSGS are mapped in Figures 5 and 6, respectively. The patterns of activations are similar between stimulations. For activations in acupuncture (Figure 5(a)), the dominant results were “stronger,” mainly including the brainstem, inferior frontal gyrus, precentral gyrus, cingulate gyrus, SI, postcentral gyrus and IPL, thalamus, insula, superior temporal gyrus, and declive of the cerebellum. The “weaker,” “disappeared,” and “arisen” regions were few and were located around the “stronger” regions. For activations in tactile stimulation (Figure 5(b)), the dominant results were “weaker” and “disappeared.” These regions mainly included the brainstem, inferior frontal gyrus, middle frontal gyrus, precentral gyrus, SI, postcentral gyrus and IPL, thalamus, insula, putamen, superior temporal gyrus and several cerebellar regions (declive, inferior semilunar lobule, pyramis, tuber, and uvula). All of these regions were bilateral. Very few regions were “stronger.” For deactivations, both stimulations were “arisen” dominant. Furthermore, the patterns of the “arisen” regions were quite similar between stimulations, including the medial frontal gyrus, middle frontal gyrus, superior frontal gyrus, precentral gyrus, SI, superior parietal lobule (SPL), posterior cingulate, precuneus, parahippocampal gyrus, cuneus, and middle occipital gyrus. For tactile stimulation, the culmen of the cerebellum was also “arisen.” Parts of the precentral gyrus, SI, SPL, precuneus, and cuneus were “stronger” in the center of “arisen” regions for tactile stimulation. Detailed results of brain regions in conjunctional maps including BA indices and sizes are summarized in Tables 3 and 4. Figure S3 shows similar results at different thresholds (*P* < 0.00001, uncorrected, and *P* < 0.01, uncorrected, resp.).

### 3.6. Nonspecific and Specific BOLD Responses Evoked by Acupuncture Stimulation (Figure 7, Figure S4, and Table 5)

**Figure 7:**
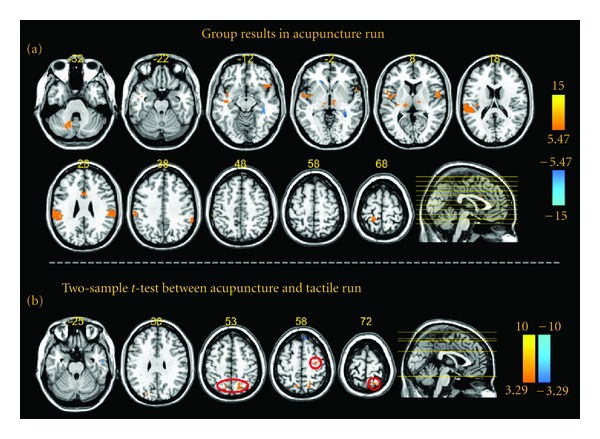
Map of the REM group results for the acupuncture run and two-sample *t*-test results between the acupuncture and tactile runs for NO PSGS. (a) indicates the REM group results evoked by acupuncture stimulation at *P* < 0.00001, uncorrected with 5 contiguous voxels. (b) shows the between-group results of “acupuncture-tactile” at *P* < 0.001, uncorrected with 5 contiguous voxels.

The group results for the one-sample *t*-test for NO PSGS in acupuncture are shown in Figure 7(a). Significant activations were present in the bilateral brainstem, the ipsilateral inferior frontal gyrus, the bilateral precentral gyrus, the contralateral cingulate gyrus, the bilateral SI, the contralateral postcentral gyrus, the bilateral IPL, the bilateral thalamus, the bilateral insula, the contralateral claustrum, the bilateral superior temporal gyrus and the contralateral dentate, and the declive, pyramis, and uvula of the cerebellum. The two-sample *t*-test results between acupuncture and tactile stimulation for NO PSGS are shown in Figure 7(b). Compared to tactile stimulation, the BOLD responses of the ipsilateral precentral gyrus, the bilateral SPL, and the bilateral precuneus were more activated in acupuncture stimulation. Detailed results including coordinates, maximum *t*-values, and sizes are summarized in Table 5. Figure S4 shows similar results at other thresholds (*P* < 0.001, uncorrected for a one-sample *t*-test and *P* < 0.01, uncorrected for a two-sample *t*-test). 

### 3.7. *De-qi*'s Influence on Our Results

Considering that *De-qi* is associated with a remedial mechanism of acupuncture [3, 28], the relationship between individual responses and *De-qi* sensations should be elucidated. No direct correlations were shown between the VAS index and the number of activations or deactivations, as well as between the VAS index and the “AVER-WR-RV *R*-value” (all *P* > 0.05), which were consistent with our previous study [11]. 

## 4. Discussion 

The present study first examined the underlying validity of using PSGS in acupuncture fMRI studies. Our results demonstrated that the orthogonality assumption was violated and prominent changes between acupuncture-evoked BOLD responses for NO PSGS and PSGS were shown in most subjects. Secondly, the impact of PSGS on the BOLD responses evoked by acupuncture stimulation was evaluated. For most subjects and the group results, the positive-correlation-dominant BOLD responses were changed into negative-correlation-dominant by PSGS, which was the nonspecific impact of PSGS for both acupuncture and tactile stimulations. In particular, our results implicated that extensive deactivations found in acupuncture fMRI studies may be the effect of global normalization other than a plausible acupuncture-specific effect. In addition, the specific impact of PSGS on acupuncture was that the statistical power of several regions in the somatosensory-related brain network was increased for PSGS, which had the most significant activations for NO PSGS. Finally, our results indicated that the “on-off”-based central BOLD responses during *De-qi* evoked by acupuncture stimulation were nonspecifically activation-dominant and were mainly around the somatosensory-related brain network. 

### 4.1. Global Normalization Is Unjustified for Exploring BOLD Responses Evoked by Acupuncture Stimulation

Many previous studies demonstrated that adverse consequences were generated by global normalization when the orthogonality assumption was violated [13, 14, 22, 24, 29]. Therefore, researchers suggested that it was necessary to examine the orthogonality assumption before the application of global normalization. First, global normalization should not be applied on the condition that the “GS-RV *R*-value” is large [22]. Second, it should not be applied on the condition that global normalization significantly changes the results [12, 13, 24], even with a small “GS-RV *R*-value” [14]. Our results for acupuncture showed that the larger the “GS-RV *R*-value,” the stronger the relationship of the experimental design and the BOLD signal changes in the whole brain (Figure 2(a)) and the more serious the impact of global normalization (Figure 2(c)). These dependences had a linear relation. The “GS-RV *R*-values” of many subjects were significant (Figure 2, Figure S1, and Table S1). Significant changes between BOLD responses for NO PSGS and PSGS were demonstrated, mainly reversing the activation-dominant trend of BOLD responses to the deactivation-dominant ones (Figures 2 and 3, Table 1, and Tables S1 and S2). Therefore, we supposed that it is unjustified for most subjects in acupuncture studies in fMRI. In this sense, we suggest that one should take precautions when interpreting previous conclusions using global normalization.

When the orthogonality assumption was violated, the main consequences caused by PSGS were that (i) the level of activity from regions that were actually associated with the experimental paradigm would be reduced, potentially decreasing statistical power and (ii) the level of activity from regions that were nonassociative with the experimental paradigm might manifest an apparent negative association [12, 14, 22–24, 30]. Our results indicated that for subjects whose global signals were positively correlated to the reference vector, the overall level of activations was reduced, whereas deactivations increased (Figures 3 and 4, Table 3, Figure S3, and Tables S1 and S2). On the contrary, for subjects whose global signals were negatively correlated with the reference vector, the overall level of activations was significantly increased, whereas deactivations were significantly reduced by PSGS. On the group level, the improper application of global normalization in our study manifested as reducing activations and introducing artificial deactivations (Figures 2, 3, and 4, Table 1, and Tables S1 and S2) according to previous findings [13, 14, 22, 24]. As an inference, if the proportion of negative correlation (positive correlation) in a group was changed, the impact of global normalization should also be changed. Therefore, we inferred that the differences for the application of global normalization, as well as the different proportions of negative correlations in a group when global normalization was applied, may partly explain the disparate results in previous fMRI acupuncture studies. Furthermore, the amplitude of activations was consistently decreased after PSGS (Table S4), which was also in accordance with previous reports [22, 30]. We also adopted an additional tactile study to examine the robustness of the current findings. The results drew similar conclusions, suggesting that they were just the fatal influence of a data processing pipeline.

### 4.2. Extensive Deactivations Introduced by Global Normalization Are Nonspecific Spurious Effects

The role of BOLD deactivation induced by acupuncture is a pivotal but controversial topic [10, 31]. A series of papers believed that the extensive deactivations in the LPNN, highly overlapped with DMN, were specific BOLD responses during *De-qi* evoked by acupuncture stimulation [3, 5, 19, 21, 31–33], which may reflect the neuroregulation mechanism of acupuncture [3, 5, 32]. However, more fMRI-based acupuncture studies failed to discover the significantly extensive deactivations [4, 7, 8, 10, 34–42]. Our results demonstrated that the central BOLD responses during *De-qi* evoked by acupuncture stimulation are activation-dominant (Figures 3 and 7, Table 5, and Table S3). For NO PSGS, no deactivations in grey matter were shown at *P* < 0.00001, uncorrected (Figure 7, Table 5), and very few deactivations were shown at *P* < 0.001, uncorrected (Figure 6 and Figure  S4). For PSGS, few deactivations were significant at *P* < 0.00001, uncorrected (Figure S3), while a great amount of deactivations were significant at *P* < 0.001, uncorrected (Figure 6). These deactivations were mainly at the occipital cortex and around the DMN. Comparing these results between acupuncture and tactile stimulation, we found that the pattern of deactivations was similar (Figure 6, Figure S3, and Table 4). These results are in accordance with findings in previous studies [13, 14, 22, 24]. Taken together, we inferred that the significant deactivations in acupuncture were spurious effects due to incorrect global normalization.

The deactivations in DMN were found in many fMRI studies across various fields [43–48]. Recent studies about the origins of the deactivations have demonstrated that deactivations in the DMN merely reflected the temporary suspension of activity of the semantic-related network by an externally passive task with reallocation of attentional resources, rather than a specific BOLD response pattern evoked by a task [49, 50]. Interestingly, global normalization often surrounded deactivations in the DMN in fMRI during the early stage [12–14, 29, 30], because these deactivations could be enhanced by incorrect global normalization [22]. Reviewing studies about deactivation in acupuncture in fMRI, we found that most of them adopted global normalization, but none of them discussed its impact for introducing deactivations [3, 5, 15, 19, 20]. Consequently, we conjectured that most deactivations reported in previous acupuncture fMRI studies were probably nonspecific manifestations of the DMN under an externally passive task [10]. Moreover, since the BOLD responses evoked by acupuncture stimulation were activation-dominant, the deactivations in the DMN were enhanced after incorrect global normalization.

### 4.3. Heightened Effects of the Most Significant Activations Produced by Global Normalization Are Specific for Acupuncture

Although most impacts of PSGS are consistent and similar between acupuncture and tactile stimulation, remarkably specific results for acupuncture produced by PSGS were shown, that is, the “stronger” activations in several regions of the somatosensory-related brain network, which were the most significant activations in both acupuncture and tactile stimulation groups for NO PSGS (Figure 6(a) and Table 3). The “stronger” activations in these regions indicated that their group-level statistical power for the acupuncture group was enhanced by PSGS. However, these regions' activations were “weaker” or “disappeared” in the tactile group (Figure 6(b) and Table 3). For tactile stimulation, all measures indicated that PSGS decreased the activations, which is similar to previous studies [13, 14, 22, 24]. In contrast, the heightened effect of the activations in these regions seemingly contradicted the reduction of the activations on the overall level and the amplitudes of BOLD signal changes for acupuncture. In fact, this heightened effect was also consistent with findings in previous studies [22, 51]. Zarahn et al. proposed that despite the reduced activations' strength, the inclusion of the global regressor might still affect a reduction in otherwise unexplained variance large enough to cause a net increase in sensitivity [51]. We inferred that the processing of PSGS would more effectively reduce the experimental-paradigm-unrelated variations in the acupuncture group rather than in the tactile group. Furthermore, we supposed that the proportion of experimental-paradigm-unrelated variations of the BOLD signals during acupuncture stimulation might be significantly larger than those during tactile stimulation, which may reflect the complexity of the underlying characteristics of BOLD responses in acupuncture.

Based on the one-sample *t*-test, our results indicate that the “on-off”-modal central BOLD responses during *De-qi* evoked by acupuncture stimulation were activation-dominant and nonspecific, mainly in the somatosensory, motor and sensory integration brain network (Figure 7, Figure S4, and Table 5). However, this does not mean that there is no specific effect of acupuncture on the central nervous system. We suggested that exploration of un-“on-off”-modal variations may capture the specific BOLD responses evoked by acupuncture stimulation. For this purpose, the novel experimental design including several conditions in a single run based on better quantification of the acupuncture stimulation and the subjective sensation should be adopted [2, 4]. Besides, the application of data-driven methods may lead to a more comprehensive understanding of the underlying mechanism of acupuncture [52–56]. As recommended recently, pattern-based approaches and statistical inferences should be widely adopted in future fMRI-based acupuncture studies [57–59].

### 4.4. Limitations

This paper still has space for improvement in upcoming studies. Firstly, due to the limitation of length, only the method of PSGS was detailed, which was more popular in previous acupuncture studies in fMRI. We also checked the results of global normalization as an additive term in the GLM model in partial datasets and acquired similar results (figures not shown). Secondly, maps and tables under the corrected threshold are not shown due to the unequally absolute *t*-values among groups. The conservative threshold in this paper is *P* < 0.00001, uncorrected, which is parallel to the *t*-values of *P* < 0.05, FWE corrected. Actually, most results in this paper were gradually changed and became independent of the threshold. Finally, all of the results, conclusions, and inferences limited to the “on-off” experimental modal detections using other paradigms should also be covered. 

## 5. Conclusions

Taken together, the present study demonstrated that the risks and detrimental consequences regarding the standard use of global normalization in fMRI are particularly relevant to acupuncture research. This preprocessing step might be unjustified for acupuncture studies in fMRI. Particularly, extensive deactivations induced by acupuncture might be the nonspecific pernicious consequences of global normalization, which may contribute to conflicting results and interpretations in acupuncture fMRI studies. The central responses evoked by acupuncture stimulation associated with *De-qi* were nonspecifically activation-dominant and were mainly around the somatosensory-related brain network, whose statistical power was specifically enhanced by PSGS. The specific BOLD responses evoked by acupuncture stimulation may be hidden in the experimental-paradigm-unrelated variations and should be explored by data-driven methods.

## Supplementary Material

Supplementary Material include Supplemental Results, four figures and four tables

## Figures and Tables

**Table 1:** Comparison of Number of Activation/Deactivation for NO PSGS and PSGS.

			Acupuncture								Tactile				
Subjects no.		Num. of activations			Num. of deactivations			Subjects no.		Num. of activations			Num. of deactivations		
		NO PSGS	PSGS	∆	NO PSGS	PSGS	∆			NO PSGS	PSGS	∆	NO PSGS	PSGS	∆
***Sub01 ***		5955	3859	−35%	2418	2258	-7%	***Sub01 ***		137	493	**260%**	1858	776	**−58%**
***Sub02 ***		5331	4335	−19%	1224	3183	**160%**	***Sub02 ***		1011	4591	**354%**	3763	2024	−46%
***Sub03 ***		8858	1681	**−81%**	23	7094	**30743%**	***Sub03 ***		5458	2861	−48%	119	2089	**1655%**
***Sub04 ***		2067	2056	−1%	8	48	**500%**	***Sub04 ***		4598	2929	−36%	122	1269	**940%**
***Sub05 ***		1514	882	−42%	15	516	**3340%**	***Sub05 ***		669	1160	73%	391	285	−27%
***Sub06 ***		1	31	**3000%**	407	112	**−72%**	***Sub06 ***		3028	2299	−24%	634	1480	**133%**
***Sub07 ***		429	426	−1%	933	666	−29%	***Sub07 ***		556	1506	**171%**	2005	1964	−2%
***Sub08 ***		8109	2422	**−70%**	18	2430	**13400%**	***Sub08 ***		10999	3706	**−66%**	49	10182	**20680%**
***Sub09 ***		640	1017	59%	61	71	16%	***Sub09 ***		7024	5328	−24%	2556	5031	97%
***Sub10 ***		9388	3007	**−68%**	3095	9423	**204%**	***Sub10 ***		2294	917	**−60%**	1	527	**52600%**
***Sub11 ***		1588	1191	−25%	447	924	**107%**	***Sub11 ***		5969	5084	−15%	2746	4033	47%
***Sub12 ***		3866	5659	46%	2090	3682	76%	***Sub12 ***		1854	1528	−18%	11	462	**4100%**
***Sub13 ***		39	6694	**17064%**	13037	2450	**−81%**	***Sub13 ***		546	499	−9%	19	75	**295%**
***Sub14 ***		228	896	**293%**	7	20	**186%**	***Sub14 ***		151	1306	**765%**	2027	1446	−29%
***Sub15 ***		5261	1849	**−65%**	381	1260	**231%**	***Sub15 ***		2200	685	**−69%**	26	164	**531%**
***Sub16 ***		278	358	29%	583	441	−24%	***Sub16 ***		9497	3009	**−68%**	813	3679	**353%**
***Sub17 ***		1043	693	−34%	138	574	**316%**	***Sub17 ***		1562	908	−42%	268	674	**151%**
***Sub18 ***		5043	1906	**−62%**	552	1863	**238%**	***Sub18 ***		9705	1959	**−80%**	0	5181	**Inf**
***Sub19 ***		1556	2972	91%	6238	4362	−30%	***Sub19 ***		2072	4972	**140%**	7039	2554	**−64%**
***Sub20 ***		6001	2295	**−62%**	684	1831	**168%**	***Sub20 ***		116	849	**632%**	227	308	36%
***Sub21 ***		360	1157	**221%**	2715	580	**−79%**	***Sub21 ***		1975	1787	−10%	530	797	50%
***Sub22 ***		570	480	−16%	1	6	**500%**	***Sub22 ***		8714	4877	−44%	1112	3986	**258%**
***Mean ***		**3097**	**2085**	**−33%**	**1594**	**1991**	**25%**	***Sub23 ***		1039	1478	42%	1052	1183	12%
								***Mean ***		**3529**	**2380**	**−33%**	**1190**	**2181**	**83%**

	***P < 0.00001 ***	**850**	**1350**	**59%**	**59**	**245**	**315%**		***P < 0.00001 ***	**2231**	**902**	**−60%**	**87**	**1486**	**1608%**
***Group ***	***P < 0.001 ***	**5831**	**4680**	**−20%**	**821**	**4722**	**475%**	***Group ***	***P < 0.001 ***	**8505**	**3509**	**−59%**	**2252**	**7520**	**234%**
	***P < 0.01 ***	**21067**	**7953**	**−34%**	**3521**	**11687**	**232%**		***P < 0.01 ***	**14955**	**6401**	**−57%**	**6098**	**14016**	**130%**

The activations and deactivations are thresholded at *P* < 0.00001, uncorrected. ∆>100% are shown in bold and colored in red (activations) or light blue (deactivations). ∆<−50% are shown in bold and colored in orange (activations) or green (deactivations). Abbreviations: ∆: (PSGS − NO PSGS)/NO PSGS∗100%.

**Table 2:** Significant brain regions of PSGS < NO PSGS.

				Acupuncture					Tactile		
Regions			Talairach		*t*-value	Voxels		Talairach		*t*-value	Voxels
		*x *	*y *	*z *			*x *	*y *	*z *		
Inferior frontal gyrus											(+95)
BA 9	L						−56	7	27	5.3	14
	R						59	10	30	4.1	8
BA 44	L						−53	10	19	4.4	16
	R						59	12	16	4.3	11
BA 45	L						−53	13	21	4.3	5
	R						56	18	5	4.2	13
BA 47	L						−53	20	−1	4.0	8
	R	42	20	−14	3.9	5	53	20	2	4.4	18
Middle frontal gyrus											(+38)
BA 6	R						−42	2	50	4.3	5
BA 9	L						−50	5	36	3.8	5
	R						53	8	36	3.9	9
Superior frontal gyrus											
BA 6	R										

Precentral gyrus											(+89)
BA 4	L						−59	−16	34	4.5	8
	R						62	−13	34	4.9	8
BA 6	L						−56	4	30	5.0	20
	R						59	0	8	4.1	6
BA 44	L						−45	0	6	4.4	14
	R						59	6	8	4.5	16

Postcentral gyrus						(+47)					(+186)
BA 1, 2, 3	L	−62	−22	31	4.1	19	−62	−19	31	5.8	42
	R	65	−22	34	3.9	6	59	−19	31	4.5	28
BA 40	L	−62	−22	18	4.2	27	−56	−23	15	6.4	21
	R						62	−25	21	5.0	12
BA 43	L						−50	−14	15	5.4	10
	R						53	−17	17	5.3	12
Inferior parietal lobule						(+51)					(+180)
BA 40	L	−62	−25	29	4.3	33	−56	−28	24	5.7	85
	R	65	−33	29	4.5	33	56	−31	26	5.1	42
Thalamus	L						−15	−11	6	4.4	47
	R						9	−11	3	3.9	10
Insula						(+6)					(+62)
BA 13	L	−53	−31	21	4.1	9	−48	−22	18	6.4	107
	R						48	−20	15	4.9	47
BA 47	R										

Claustrum	L						−33	0	8	4.5	23
	R						36	−2	8	3.7	6
Extranuclear											(+104)
Putamen	L						−27	3	0	4.0	22
	R										
Subgyral											(+80)
BA 21	L						−42	−3	−7	4.5	6
	R						42	−9	−7	3.9	3

Middle temporal gyrus											(+18)
Superior temporal gyrus						(+11)					(+158)
BA 13	L						−42	−20	9	4.3	8
BA 22	L	−56	0	3	4.0	7	−56	6	2	6.6	51
	R						59	3	5	5.7	25
BA 38	L						−42	8	−11	4.1	10
	R						45	8	−8	4.0	8
BA 41	L						−53	−20	9	5.5	19
	R										
BA 42	L	−62	−31	21	4.0	10	−62	−20	12	5.7	13
	R						56	−28	15	4.0	9
Transverse temporal gyrus											(+6)
BA 41	L						−53	−23	12	5.7	14
	R						50	−17	12	5.3	5
BA 42	L										
	R						59	−14	12	4.9	5

Cerebellum											
Declive	L						−33	−65	−19	4.3	25
	R						30	−71	−19	4.0	22
Pyramis	L						−27	−74	−32	4.6	6
	R						30	−72	−32	4.1	3
Tuber	L						−33	−74	−27	4.1	6
Uvula	L						−33	−74	−24	3.9	11

The coordination of voxel with the maximal *t* within each region is listed. The regions are thresholded at *P* < 0.001, uncorrected. The number of detected white matter is listed in the parenthesis of each region.

Abbreviations: BA: Brodmann area; L: left; R: right.

**Table 3:** Changes of activations between PSGS and NO PSGS.

Regions			Tactile				Acupuncture		
		Increased voxels no.	Decreased voxels no.	Disappeared voxels no.	Arisen voxels no.	Increased voxels no.	Decreased voxels no.	Disappeared voxels no.	Arisen voxels no.
Brainstem									
Midbrain	L		44	64		46	14	25	6
	R		11	56		15	21	23	

Inferior frontal gyrus		(9)							
BA 9, 44, 45, 46, 47	L		29 (31)	72 (94)		23 (19)	10 (14)	15 (16)	
	R		48 (61)	134 (208)		38 (57)	8 (12)	13 (22)	(16)
Medial frontal gyrus									
BA 6, 8, 32	L	5		8 (5)		7		11 (11)	
	R			16 (11)		29 (5)	5	5	
Middle frontal gyrus									
BA 6, 8, 9	L	5	12 (5)	47 (35)					
	R		18 (25)	77 (120)		22 (17)			
Superior frontal gyrus									
BA 6, 8	L		7	13 (5)				6	
	R		20 (7)	17 (35)		16	6	7 (16)	6
Precentral gyrus									
BA 4, 6, 43, 44	L	6	50 (49)	34 (23)		26 (30)	9 (22)	11 (6)	
	R	5	39 (42)	37 (30)		34 (36)	6 (12)	(6)	

Anterior cingulate									
BA 24, 33	L		18			5		(6)	
	R					5		5 (5)	
Cingulate gyrus									
BA 24, 32	L		(12)	22 (46)		55 (16)	21 (17)	9 (33)	9 (8)
	R					40 (36)	11 (6)	22 (17)	16 (5)

Postcentral gyrus									
BA 1, 2, 3	L	6	51	22		20	26	13	
	R	9	29	19		29			
BA 40, 43	L		29 (106)	14 (47)		26 (59)	(60)	12 (38)	
	R	(20)	25 (78)	10 (29)		23 (91)	(5)		
Inferior parietal lobule									
BA 40	L	11 (12)	89 (107)	77 (78)		80 (42)	16 (48)	20 (26)	
	R	5	51 (74)	33 (40)		31 (62)	53 (40)	12 (26)	
Supramarginal gyrus									
BA 40	L		5 (8)	9 (41)		5			
	R			5 (20)		5	8 (5)	(12)	
Thalamus	L		63	92		45	53	27	
	R		29	44		42	33	25	
Insula									
BA 13	L	5 (25)	108 (108)	96 (102)		107 (91)	24 (31)	20 (28)	(5)
	R	6 (18)	71 (165)	89 (110)		43 (170)	17 (21)	12 (12)	6 (14)
Claustrum	L		31	21		35	5	12	12
	R		13	31		7		9	
Extranuclear									
BA 13	L	(7)	(124)	(200)		(38)	5 (27)	(93)	(21)
	R	(10)	(37)	(308)		(133)	(16)	(48)	(21)
Caudate	R			13					
Putamen	L		80	101		88	5	17	32
	R		21	145		10	5	15	20
Lateral globus pallidus	L			17					
	R			5					
Subgyral									
BA 21	L	(16)	9 (71)	(130)		6 (17)	(14)	(50)	(10)
	R	(11)	6 (46)	(109)		5 (75)			(7)

Parahippocampal gyrus									
BA 34	L			5 (13)					
	R			5 (7)					
Amygdala	L			5					
	R			5					
Fusiform gyrus									
BA 19	R			9 (11)					
Middle temporal gyrus									
BA 21, 37, 39	L		(10)	21 (58)		(15)	(12)	6 (10)	
	R	(8)	8 (27)	38 (57)		(25)	(20)	(21)	
Superior temporal gyrus									
BA 22, 38, 41, 42	L	(5)	92 (97)	98 (133)		89 (20)	25 (13)	28 (21)	19 (9)
	R	20 (13)	50 (63)	92 (67)		43 (138)	8 (5)	12 (7)	10 (11)
Transverse temporal gyrus									
BA 41, 42	L		20 (7)			21 (4)			
	R		10	5		7 (10)			

Cerebellum									
Dentate	L						8	19	
Culmen	L			5		29	20	32	
	R			13		3		24	
Declive	L	19	71	114		120	70	74	10
	R	6	27	117		13	5	13	7
Cerebellar tonsil	L			5				16	
	R			5					
Fastigium	L							7	
Nodule	L							8	
Inferior semilunar lobule	L			41				32	
	R			35					
Pyramis	L		11	75			20	55	
	R			27					

Tuber	L		8	37				30	
Uvula	L		29	43			15	34	
	R			27					

The coordination of voxel with the maximal *t* within each region is listed. The regions are thresholded at *P* < 0.001, uncorrected. The number of detected white matter is listed in the parenthesis of each region.

Abbreviations: BA: Brodmann area; L: left; R: right.

**Table 4:** Changes of deactivations between PSGS and NO PSGS.

Regions			Tactile				Acupuncture		
		Increased voxels no.	Decreased voxels no.	Disappeared voxels no.	Arisen voxels no.	Increased voxels no.	Decreased voxels no.	Disappeared voxels no.	Arisen voxels no.
Brainstem									
Pons	L								2
Medulla	R								3

Medial frontal gyrus									
BA 6, 8, 9, 10	L				14 (44)				5 (24)
	R	6 (11)	5 (5)		37 (82)				(9)
Middle frontal gyrus									
BA 6, 8, 9	L				10 (27)				16 (13)
	R	18 (5)			59 (77)	5			38 (37)
Superior frontal gyrus									
BA 6, 8	L				12 (66)				34 (37)
	R				36 (43)	6			37 (28)
Paracentral lobule									
BA 4, 6	L				6				
	R				13 (23)				
Precentral gyrus									
BA 4, 6, 43, 44	L	19 (66)	(7)		55 (134)				(31)
	R	62 (98)			86 (133)				19 (34)

Anterior cingulate									
BA24, 33	L				7 (38)			(5)	(15)
	R				(5)				
Cingulate gyrus									
BA 24, 32	L				(14)				
	R				15 (39)				
Cingulate gyrus									
BA 31	L								18 (10)
	R								13 (32)

Postcentral gyrus									
BA 1, 2, 3	L				28 (35)				
	R	26 (13)	17 (13)		29 (38)				31
BA 40, 43	L								
	R								(16)
BA 5, 7	R				9				
Inferior parietal lobule									
BA 39	R				(12)				7 (12)
Superior parietal lobule									
BA 7	L	15 (7)			22 (12)				8 (8)
	R	63 (29)			34 (13)				33 (27)
Supramarginal gyrus									
BA 40	L								(6)
	R								(13)
Thalamus	R				5				8
Caudate	L				10				
Extranuclear									
BA 13	L	(40)	(5)	(7)	(94)		(13)	(10)	(45)
	R	(28)	(22)	(8)	(139)		(53)	(7)	(96)
Subgyral									
BA 21	L	(169)	(51)	(23)	(494)		(41)	(30)	(150)
	R	7 (200)	(105)	(71)	15 (687)		(146)	(79)	(532)

Posterior cingulate									
BA 30, 31	L				13 (30)				7 (41)
	R				(32)				15 (70)
Precuneus									
BA 7, 19, 31, 39	L	93 (73)			139 (221)				158 (203)
	R	88 (89)			148 (220)				160 (214)
Parahippocampal gyrus									
BA 19, 30, 35, 36	L				32 (69)				8 (69)
	R				8 (30)	7			52 (89)
Hippocampus	L				5				6
	R					5			11
Inferior temporal gyrus									
BA 20, 21	L								5 (16)
	R								5 (9)
Middle temporal gyrus									
BA 37, 39	L				13 (33)				5 (6)
	R	(18)			8 (114)				42 (88)
Superior temporal gyrus									
BA 22, 38, 41, 42	R								(8)
Angular gyrus									
BA 39	L								5 (28)
	R	(5)			5 (12)				10 (33)
Fusiform gyrus									
BA 20, 37	L				9 (8)				5 (7)
	R				7				20 (23)
Cuneus									
BA 17, 18, 19, 7	L	28			110 (190)				52 (98)
	R	37			112 (127)				93 (158)
Inferior occipital gyrus									
BA 19	R								6 (17)
Middle occipital gyrus									
BA 18, 19, 37	L	(6)			27 (152)				29 (42)
	R	(28)		(6)	27 (126)				82 (202)
Superior occipital gyrus									
BA 19	L	12 (8)			12 (8)				
	R				14 (13)				6 (9)
Lingual gyrus									
BA 17, 18, 19	L				11 (15)				(8)
	R				6 (13)				12 (22)

Cerebellum									
Culmen	L				110				11
	R				18				

The coordination of voxel with the maximal *t* within each region is listed. The regions are thresholded at *P* < 0.001, uncorrected. The number of detected white matter is listed in the parenthesis of each region

Abbreviations: BA: Brodmann area; L: left; R: right.

**Table 5:** Nonspecific and specific BOLD responses evoked by acupuncture stimulation.

			Acupuncture					Acupuncture > tactile			
Regions			Talairach		*t*-value	Voxels		Talairach		*t*-value	Voxels
		*x *	*y *	*z *			*x *	*y *	*z *		
Brainstem											
Midbrain	L	−15	−23	−1	5.57	5					
	R	9	−15	−2	5.97	7					

Inferior frontal gyrus											
B A47	R	42	20	−14	7.2	5					
Precentral gyrus											
BA 6, 44	L	−48	−3	6	6.4	9					
	R	56	−3	6	6.3	5	33	−9	56	3.91	6
Cingulate gyrus											
BA 24	L	−9	13	32	6.6	9					
Postcentral gyrus											
BA 2, 3	L	−18	−35	65	7.91	19					
	R	65	−22	31	6.05	5					
BA 40	L	−53	−28	21	7.46	20					
Inferior parietal lobule											
BA 40	L	−65	−31	29	8.14	51					
	R	59	−42	38	7.13	36					
Thalamus	L	9	−17	1	6.68	27					
	R	−15	−20	7	6.4	44					
Insula											
BA 13	L	−50	−31	18	7.21	32					
Claustrum											
	L	−36	0	3	6.09	8					
Superior parietal lobule											
BA 7	L						−12	−61	56	4.11	3
	R						21	−50	58	3.73	12
Precuneus											
BA 7, 19	L						−9	−58	58	3.97	11
	R						18	−56	53	3.77	8
Superior temporal gyrus											
BA 22, 38, 41, 42	L	−59	−34	21	6.57	38					
	R	59	9	−3	6.51	7					

Cerebellum											
Dentate	L	−15	−62	−25	8.19	11					
Declive	L	−18	−65	−22	7.37	28					
Pyramis	L	−15	−65	−27	6.81	5					
Uvula	L	−18	−68	−24	6.48	5					

The coordination of voxel with the maximal *t* within each region is listed. The regions are thresholded at *P* < 0.00001, uncorrected for “acupuncture” and at *P* < 0.001, uncorrected for “acupuncture > tactile.”

Abbreviations: BA: Brodmann area; L: left; R: right.
