# Supplementary material for: Impact of Global Normalization in fMRI Acupuncture Studies
Source: Evid Based Complement Alternat Med. 2012 Dec 17;2012:467061. doi: 10.1155/2012/467061 (PMC3534243; doi:10.1155/2012/467061)
Supplement: Supplementary file 1 — Supplementary Material include Supplemental Results, four figures and four tables [file 467061.f1.pdf]

Supplementary Materials

Results

The amplitudes of BOLD signal changes (ABSC) for PSGS and NO PSGS are listed in TableS4. Brain regions which are involved in the ‘stronger’ and ‘weaker’ activations of the conjunction analyses maps were tested. The table shows that after PSGS, the ABSCs of all of these regions were decreased. For each region, the ABSC evoked by acupuncture stimulation was slightly less than that evoked by tactile stimulation for both NO PSGS and PSGS. The ABSC in the ipsilateral direction was slightly less than that in the contralateral direction for both stimulations and for both NO PSGS and PSGS. The maximum for both stimulations was at the contralateral postcentral gyrus (BA40, 43), 0.37% for PSGS and 0.41% for NO PSGS in acupuncture and 0.49% for PSGS and 0.54% for NO PSGS in tactile stimulation.

Figure Captions

FigureS1. Correlation between the reference vector (RV) and the global signal (GS) in the acupuncture run. Panel C indicates the GS-RV R-value of each subject. The two dashed lines represent the threshold of the R-values at $p < 0.05$, uncorrected. The other panels show the RV and GS of the representative subjects.

FigureS2. Correlation between the reference vector (RV) and the global signal (GS) in the tactile run. Panel C indicates the GS-RV R-value of each subject. The two dashed lines represent the threshold of the R-values at $p < 0.05$, uncorrected. The other panels show the RV

and GS of the representative subjects.

FigureS3. Changes in activations/deactivations between NO PSGS and PSGS. The map is based on the REM group results at $p < 0.00001$, uncorrected and $p < 0.01$, uncorrected respectively. Panel A shows the changes in the acupuncture run and Panel B shows those in the tactile run. For each panel, the meaning for each color is identical to that in Figure5 and Figure6.

FigureS4. Map of the REM group results in the acupuncture run and two sample *t*-test results between the acupuncture and tactile run for NO PSGS. Panel A indicates the REM group results evoked by acupuncture stimulation at $p<0.001$, uncorrected with 5 contiguous voxels. Panel B shows the between group results of ‘acupuncture – tactile’ at $p<0.01$, uncorrected with 5 contiguous voxels.

FigureS1

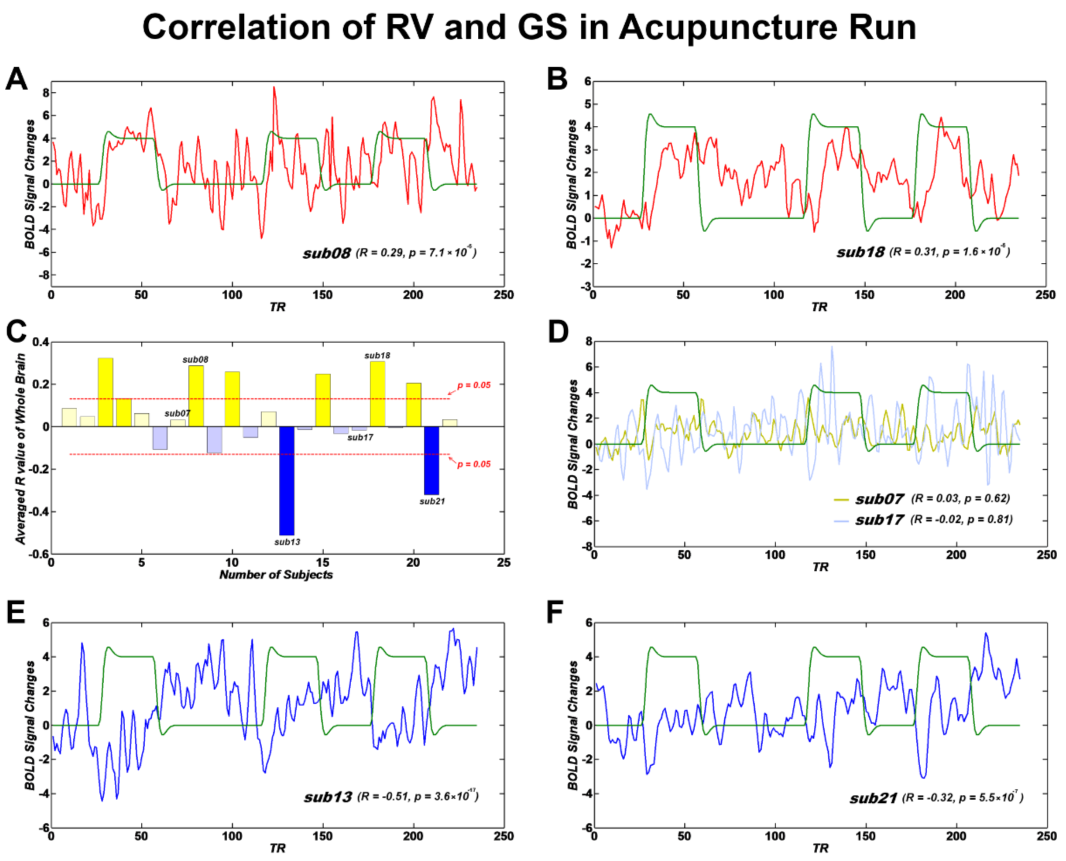
Correlation of RV and GS in Acupuncture Run
A
B
C
D
E
F
BOLD Signal Changes
TR
sub08 (R = 0.29, p = 7.1 × 10^-6)
sub18 (R = 0.31, p = 1.6 × 10^-6)
Averaged R value of Whole Brain
Number of Subjects
p = 0.05
sub08
sub18
sub07
sub17
sub13
sub21
sub07 (R = 0.03, p = 0.62)
sub17 (R = -0.02, p = 0.81)
sub13 (R = -0.51, p = 3.6×10^-17)
sub21 (R = -0.32, p = 5.5×10^-7)

FigureS2

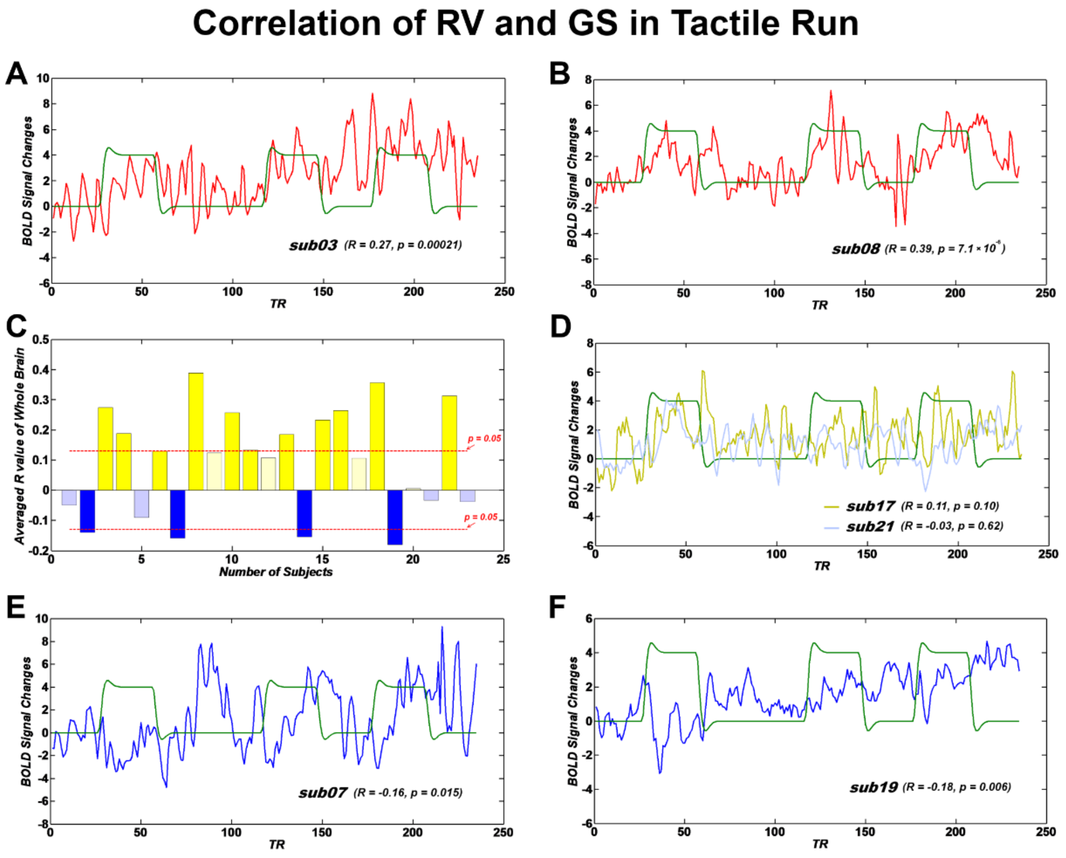
Correlation of RV and GS in Tactile Run
A
BOLD Signal Changes
TR
sub03 (R = 0.27, p = 0.00021)
B
BOLD Signal Changes
TR
sub08 (R = 0.39, p = 7.1 × 10^-5)
C
Averaged R value of Whole Brain
Number of Subjects
p = 0.05
p = 0.05
D
BOLD Signal Changes
TR
sub17 (R = 0.11, p = 0.10)
sub21 (R = -0.03, p = 0.62)
E
BOLD Signal Changes
TR
sub07 (R = -0.16, p = 0.015)
F
BOLD Signal Changes
TR
sub19 (R = -0.18, p = 0.006)

FigureS3

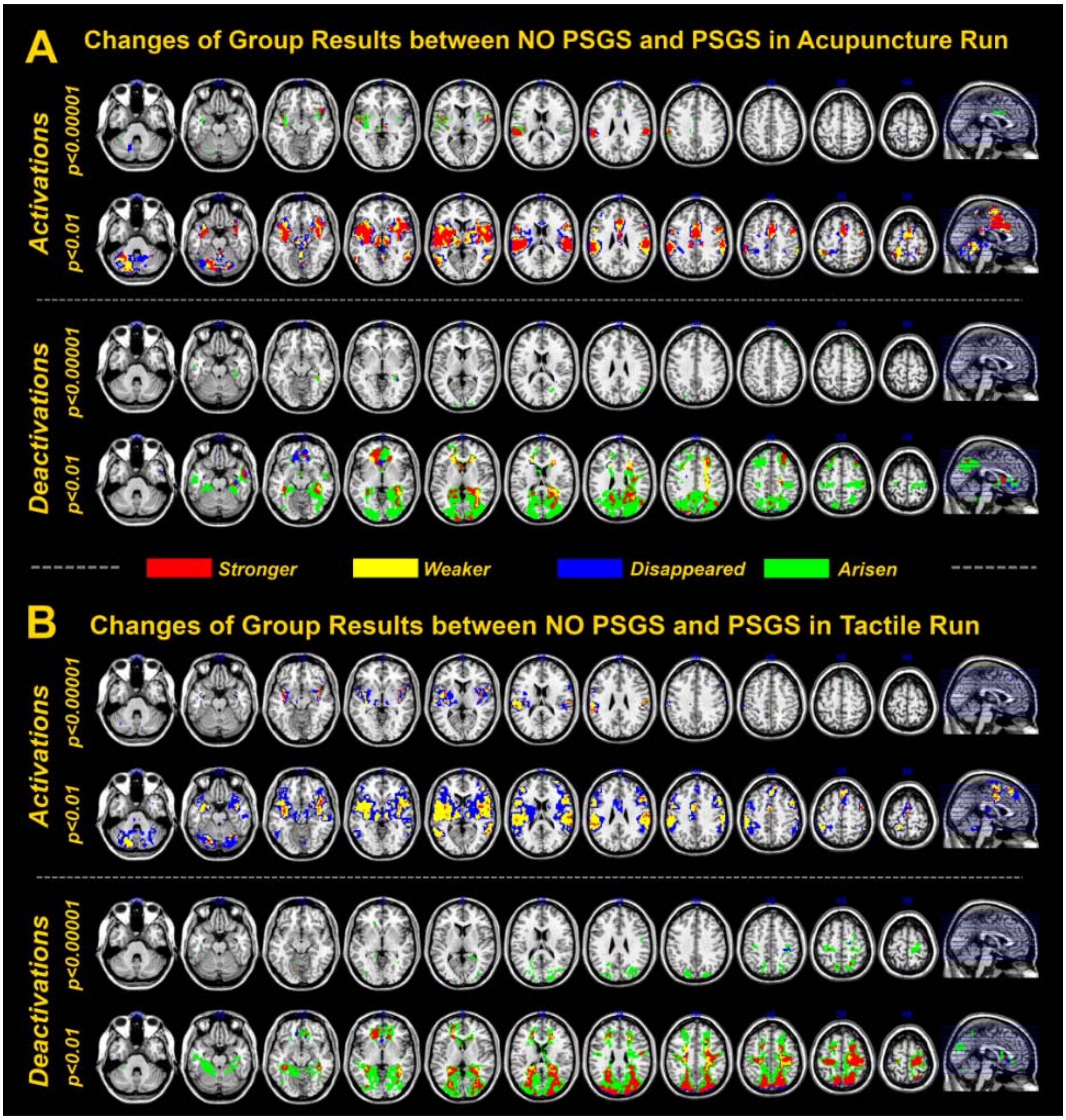
A Changes of Group Results between NO PSGS and PSGS in Acupuncture Run
Activations
p<0.00001
p<0.01
Deactivations
p<0.00001
p<0.01
Stronger
Weaker
Disappeared
Arisen
B Changes of Group Results between NO PSGS and PSGS in Tactile Run
Activations
p<0.00001
p<0.01
Deactivations
p<0.00001
p<0.01

FigureS4

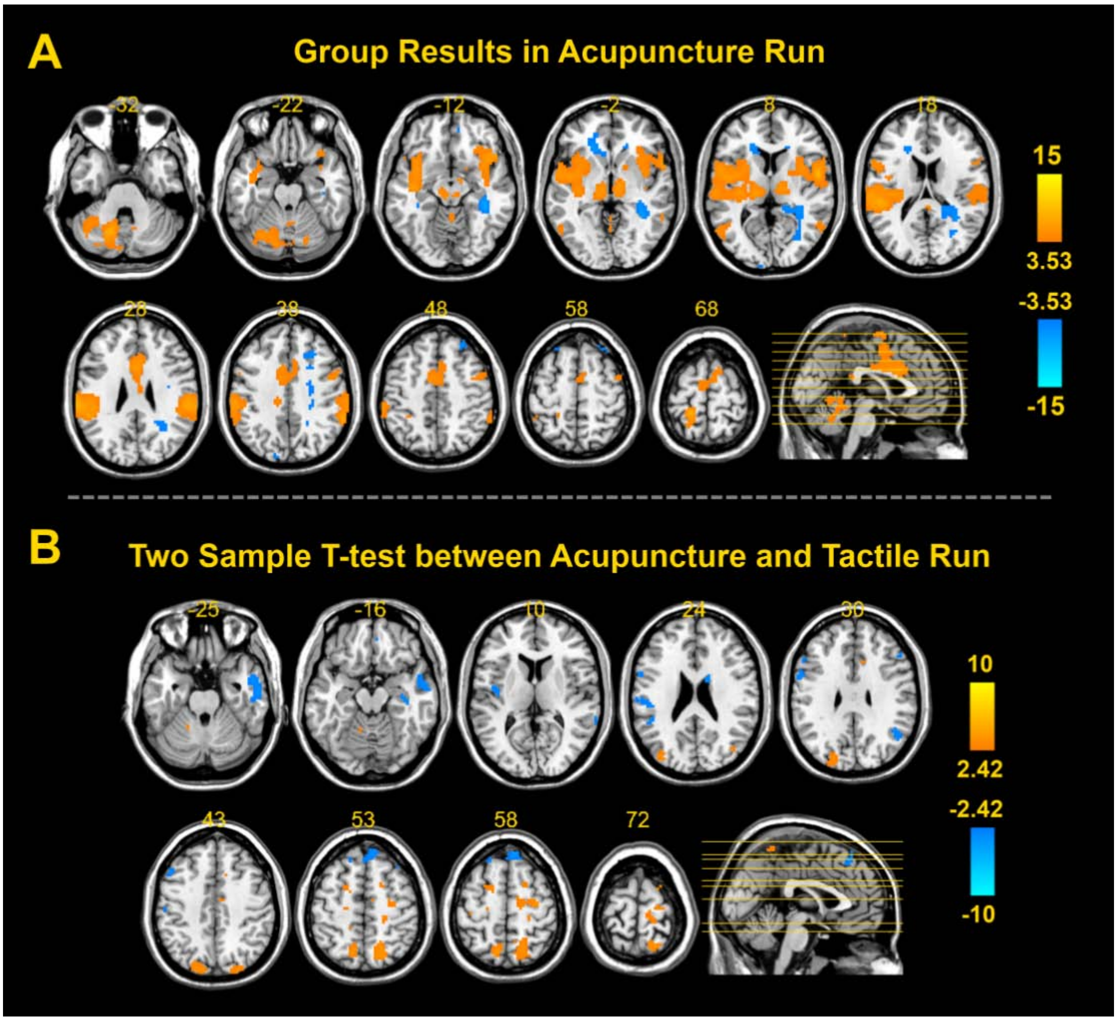

A
Group Results in Acupuncture Run
-32
-22
-12
-2
8
18
15
3.53
-3.53
-15
28
38
48
58
68
B
Two Sample T-test between Acupuncture and Tactile Run
-25
-16
10
24
36
10
2.42
-2.42
-10
43
53
58
72

Table S1. Comparison of Averaged R-value for NO PSGS and PSGS

Acupuncture

Number of subjects	Averaged R-value of Whole Brain		
	NO PSGS	PSGS	PSGS - NO PSGS
sub01	0.062	0.003	-0.059
sub02	0.007	-0.021	**-0.028**
sub03	0.165	-0.042	**-0.207**
sub04	0.072	-0.019	**-0.091**
sub05	0.036	-0.004	**-0.04**
sub06	-0.052	0.015	**0.067**
sub07	0.024	0.014	-0.01
sub08	0.136	-0.021	**-0.157**
sub09	-0.063	0.013	**0.076**
sub10	0.083	-0.057	**-0.14**
sub11	-0.022	0.002	**0.024**
sub12	0.024	-0.003	**-0.027**
sub13	-0.225	0.050	**0.275**
sub14	-0.014	-0.005	0.009
sub15	0.130	-0.015	**-0.145**
sub16	-0.008	0.001	**0.009**
sub17	-0.009	-0.004	0.005
sub18	0.085	-0.037	**-0.122**
sub19	-0.010	0.005	**0.015**
sub20	0.089	-0.012	**-0.101**
sub21	-0.139	0.019	**0.158**
sub22	0.016	0.000	-0.016
mean	0.018	-0.005	**-0.023**
Paired t-test			**p = 2.3301e-005 (t = 5.11)**

Tactile

Number of subjects	Averaged R-value of Whole Brain		
	NO PSGS	PSGS	PSGS - NO PSGS
sub01	-0.035	0.004	**0.039**
sub02	-0.037	0.037	**0.074**
sub03	0.098	-0.041	**-0.139**
sub04	0.056	-0.019	**-0.075**
sub05	-0.033	0.004	**0.037**
sub06	0.042	-0.027	**-0.069**
sub07	-0.067	0.023	**0.09**
sub08	0.137	-0.043	**-0.18**
sub09	0.024	-0.013	**-0.037**
sub10	0.107	-0.039	**-0.146**
sub11	0.055	-0.009	**-0.064**
sub12	0.059	-0.015	**-0.074**
sub13	0.096	-0.024	**-0.12**
sub14	-0.075	0.019	**0.094**
sub15	0.091	-0.013	**-0.104**
sub16	0.105	-0.028	**-0.133**
sub17	0.044	-0.009	**-0.053**
sub18	0.148	-0.045	**-0.193**
sub19	-0.046	0.027	**0.073**
sub20	-0.013	-0.007	0.006
sub21	-0.03	-0.014	0.016
sub22	0.132	-0.024	**-0.156**
sub23	-0.019	0.000	**0.019**
mean	0.036	-0.011	**-0.048**
Paired t-test			**p = 3.5567e-008 (t = 7.91)**

Averaged R-value for the whole brain whose sign is reversed in PSGS as shown in bold. Before the paired t-test values of both NO PSGS and PSGS of a subject are reversed if the value of NO PSGS is negative.

Table S2. Comparison of Number of Activation/Deactivation for NO PSGS and PSGS

Acupuncture

Subjects Num.	Num. of Activations			Num. of Deactivations		
	NO PSGS	PSGS	△	NO PSGS	PSGS	△
sub01	16536	12100	-27%	8035	8770	9%
sub02	12815	9933	-22%	12060	18089	50%
sub03	31146	5757	**-82%**	321	17782	**5440%**
sub04	7524	5782	-23%	482	2969	**516%**
sub05	11223	4662	**-58%**	694	8781	**1165%**
sub06	64	4211	**6480%**	7256	1538	**-79%**
sub07	7093	5237	-26%	2910	2658	-9%
sub08	26323	8132	**-69%**	754	14987	**1888%**
sub09	3060	5635	84%	5238	2931	-44%
sub10	25675	8277	**-68%**	6175	22172	**259%**
sub11	8319	5344	-36%	5983	9332	56%
sub12	14318	14503	1%	7748	11688	51%
sub13	769	20781	**2602%**	34899	8052	**-77%**
sub14	2223	4198	89%	790	2154	**173%**
sub15	24058	8418	**-65%**	1846	8343	**352%**
sub16	2319	3216	39%	6669	4370	-34%
sub17	7455	5041	-32%	2287	6715	**194%**
sub18	17271	6221	**-64%**	2026	12740	**529%**
sub19	6687	10596	58%	14237	9681	-32%
sub20	19808	7895	**-60%**	2740	9533	**248%**
sub21	1953	6912	**254%**	21059	4313	**-80%**
sub22	5150	3265	-37%	636	1853	**191%**
Mean	**11445**	**7551**	**-34%**	**6584**	**8611**	**31%**

Tactile

Subjects Num.	Num. of Activations			Num. of Deactivations		
	NO PSGS	PSGS	△	NO PSGS	PSGS	△
sub01	1794	5805	**224%**	14946	5734	**-62%**
sub02	5027	18583	**270%**	12249	6000	**-51%**
sub03	16674	8097	**-51%**	2407	14404	**498%**
sub04	13195	7919	-40%	3742	12075	**223%**
sub05	3531	5520	56%	7414	4621	-38%
sub06	9142	6419	-30%	5964	11258	89%
sub07	4410	9928	**125%**	11910	7748	-35%
sub08	27111	8718	**-68%**	1358	22964	**1591%**
sub09	15444	10906	-29%	8740	14718	68%
sub10	15665	4847	**-69%**	236	12608	**5242%**
sub11	13989	10695	-24%	8924	12023	35%
sub12	10080	5443	-46%	636	7129	**1021%**
sub13	5509	2601	**-53%**	166	2075	**1150%**
sub14	2696	7674	**185%**	13499	6516	**-52%**
sub15	14785	4528	**-69%**	609	4318	**609%**
sub16	24116	7481	**-69%**	2581	16091	**523%**
sub17	9415	4423	**-53%**	1996	5881	**195%**
sub18	32003	6136	**-81%**	185	19206	**10282%**
sub19	7321	16711	**128%**	18580	7451	**-60%**
sub20	2334	5454	**134%**	6028	5589	-7%
sub21	7685	6496	-15%	8057	9275	15%
sub22	20326	10068	**-50%**	4847	15963	**229%**
sub23	3989	4879	22%	5700	5006	-12%
Mean	**11576**	**7797**	**-33%**	**6121**	**9941**	**66%**

The activations and deactivations are thersholded at $p<0.01$, uncorrected. △>100% are shown in bold and colored in red (activations) or light blue (deactivations). △<-50% are shown in bold and colored in orange (activations) or green (deactivations). △ = (PSGS - NO PSGS)/NO PSGS*100%.

Table S3. Comparison of Ratio of Activation/Deactivation for NO PSGS and PSGS

Acupuncture

Subjects Num.	NO PSGS			PSGS		
	Ac Num.	De Num.	Ratio	Ac Num.	De Num.	Ratio
sub01	5955	2418	246%	3859	2258	171%
sub02	5331	1224	436%	4335	3183	136%
sub03	8858	23	38513%	1681	7094	24%
sub04	2067	8	25838%	2056	48	4283%
sub05	1514	15	10093%	882	516	171%
sub06	1	407	0.25%	31	112	28%
sub07	429	933	46%	426	666	64%
sub08	8109	18	45050%	2422	2430	100%
sub09	640	61	1049%	1017	71	1432%
sub10	9388	3095	303%	3007	9423	32%
sub11	1588	447	355%	1191	924	129%
sub12	3866	2090	185%	5659	3682	154%
sub13	39	13037	0.30%	6694	2450	273%
sub14	228	7	3257%	896	20	4480%
sub15	5261	381	1381%	1849	1260	147%
sub16	278	583	48%	358	441	81%
sub17	1043	138	756%	693	574	121%
sub18	5043	552	914%	1906	1863	102%
sub19	1556	6238	25%	2972	4362	68%
sub20	6001	684	877%	2295	1831	125%
sub21	360	2715	13%	1157	580	199%
sub22	570	1	57000%	480	6	8000%
Mean	**3097**	**1594**	**194%**	**2085**	**1991**	**105%**
Mean_0.01	**11445**	**6584**	**174%**	**7551**	**8611**	**88%**

Tactile

Subjects Num.	NO PSGS			PSGS		
	Ac Num.	De Num.	Ratio	Ac Num.	De Num.	Ratio
sub01	137	1858	7%	493	776	64%
sub02	1011	3763	27%	4591	2024	227%
sub03	5458	119	4587%	2861	2089	137%
sub04	4598	122	3769%	2929	1269	231%
sub05	669	391	171%	1160	285	407%
sub06	3028	634	478%	2299	1480	155%
sub07	556	2005	28%	1506	1964	77%
sub08	10999	49	22447%	3706	10182	36%
sub09	7024	2556	275%	5328	5031	106%
sub10	2294	1	229400%	917	527	174%
sub11	5969	2746	217%	5084	4033	126%
sub12	1854	11	16855%	1528	462	331%
sub13	546	19	2874%	499	75	665%
sub14	151	2027	7%	1306	1446	90%
sub15	2200	26	8462%	685	164	418%
sub16	9497	813	1168%	3009	3679	82%
sub17	1562	268	583%	908	674	135%
sub18	9705	0	Inf	1959	5181	38%
sub19	2072	7039	29%	4972	2554	195%
sub20	116	227	51%	849	308	276%
sub21	1975	530	373%	1787	797	224%
sub22	8714	1112	784%	4877	3986	122%
sub23	1039	1052	99%	1478	1183	125%
Mean	**3529**	**1190**	**297%**	**2380**	**2181**	**109%**
Mean_0.01	**11576**	**6121**	**189%**	**7797**	**9941**	**78%**

Group	*p<0.00001*	**850**	**59**	**1441%**	**1350**	**245**	**551%**
	p<0.001	**5831**	**821**	**710%**	**4680**	**4722**	**99%**
	P<0.01	**21067**	**3521**	**598%**	**7953**	**11687**	**68%**

Group	*p<0.00001*	**2231**	**87**	**2564%**	**902**	**1486**	**61%**
	p<0.001	**8505**	**2252**	**378%**	**3509**	**7520**	**47%**
	P<0.01	**14955**	**6098**	**245%**	**6401**	**14016**	**46%**

The activations and deactivations are thresholded at p<0.00001, uncorrected. The mean value across subjects and the value at the group level are shown in bold. Mean_0.01 represents the mean value across subjects at p<0.01, uncorrected. Ratio = Ac Num./De Num.*100%. Abbreviations: Ac-activations; De-deactivations

TableS4. Amplitude of BOLD signal changes between NO PSGS and PSGS

Regions		Acupuncture			Tactile		
		NO PSGS	PSGS	Size	NO PSGS	PSGS	Size
Brainstem							
Midbrain	L	0.15±0.02	0.11±0.02	60	0.16±0.02	0.11±0.03	45
	R	0.17±0.02	0.13±0.02	36	0.12±0.02	0.06±0.02	12
Inferior Frontal Gyrus							
BA9, 13, 44, 45, 47	L	0.22±0.05	0.18±0.05	39	0.33±0.07	0.28±0.09	29
	R	0.24±0.09	0.20±0.09	54	0.24±0.07	0.19±0.08	54
Medial Frontal Gyrus							
BA6, 8, 32	L	0.19±0.04	0.15±0.04	11	0.28±0.09	0.24±0.09	7
	R	0.21±0.05	0.17±0.05	35	0.28±0.08	0.23±0.09	16
Middle Frontal Gyrus							
BA6, 8, 9	L						
	R	0.26±0.05	0.22±0.05	28	0.24±0.05	0.19±0.06	28
Superior Frontal Gyrus							
BA6	L	0.19±0.02	0.15±0.02	5	0.18±0.01	0.12±0.02	8
	R	0.21±0.04	0.17±0.04	22	0.21±0.04	0.16±0.05	25
Precentral Gyrus							
BA4, 6, 43, 44	L	0.25±0.08	0.21±0.08	40	0.35±0.11	0.30±0.12	60
	R	0.22±0.04	0.18±0.04	45	0.25±0.06	0.20±0.07	47
Anterior Cingulate							
BA24	L	0.20±0.03	0.15±0.03	8			
	R	0.17±0.03	0.13±0.03	6			
Cingulate Gyrus							
BA24, 32	L	0.20±0.05	0.16±0.05	79	0.21±0.02	0.16±0.03	23
	R	0.21±0.05	0.17±0.05	53			
Postcentral Gyrus							
BA1, 2, 3	L	0.30±0.09	0.26±0.09	50	0.40±0.12	0.34±0.12	60
	R	0.33±0.07	0.29±0.07	36	0.36±0.09	0.30±0.09	36
BA5, 7	L	0.18±0.02	0.14±0.02	9			
	R						
BA40, 43	L	0.41±0.11	0.37±0.11	29	0.54±0.15	0.49±0.15	31
	R	0.30±0.06	0.26±0.06	23	0.37±0.07	0.31±0.08	29
Inferior Parietal Lobule							
BA40	L	0.29±0.09	0.25±0.09	96	0.34±0.10	0.29±0.11	100
	R	0.32±0.09	0.28±0.09	84	0.37±0.08	0.32±0.08	56
Supramarginal Gyrus							
BA40	L	0.23±0.02	0.19±0.02	7	0.24±0.06	0.19±0.08	5
	R	0.25±0.04	0.21±0.04	13			
Thalamus							
	L	0.17±0.04	0.13±0.04	98	0.19±0.04	0.13±0.05	63

	R	0.18±0.04	0.14±0.04	75	0.15±0.04	0.09±0.04	30
Insula							
BA13	L	0.22±0.07	0.17±0.07	141	0.28±0.08	0.23±0.08	117
	R	0.19±0.03	0.15±0.03	73	0.21±0.05	0.16±0.05	82
Lentiform Nucleus							
	L	0.14±0.02	0.10±0.02	94	0.13±0.01	0.08±0.01	86
	R	0.13±0.02	0.09±0.02	14	0.12±0.01	0.07±0.01	21
Claustrum							
	L	0.18±0.02	0.14±0.12	40	0.21±0.03	0.15±0.04	32
	R	0.16±0.03	0.12±0.03	8	0.14±0.01	0.08±0.01	13
Extra-Nuclear							
BA13	L	0.25±0.02	0.21±0.02	6			
	R	0.21±0.03	0.17±0.03	9	0.23±0.03	0.19±0.04	6
Sub-Gyral							
BA21	L	0.24±0.03	0.20±0.03	11	0.24±0.03	0.19±0.03	10
	R	0.16±0.01	0.12±0.01	5	0.19±0.01	0.13±0.01	6
Middle Temporal Gyrus							
BA21, 37, 39	L	0.20±0.02	0.16±0.02	10			
	R	0.24±0.03	0.20±0.03	11	0.33±0.04	0.29±0.06	11
Superior Temporal Gyrus							
BA22, 38, 41, 42	L	0.27±0.10	0.23±0.10	121	0.38±0.14	0.32±0.14	89
	R	0.28±0.07	0.24±0.07	53	0.31±0.10	0.26±0.11	72
Transverse Temporal Gyrus							
BA41, 42	L	0.29±0.08	0.25±0.08	24	0.39±0.12	0.37±0.15	20
	R	0.29±0.07	0.25±0.07	7	0.33±0.07	0.27±0.07	10
Cerebellum							
Culmen	L	0.17±0.03	0.13±0.03	49			
	R	0.14±0.01	0.10±0.01	5			
Declive	L	0.19±0.04	0.15±0.04	190	0.19±0.03	0.13±0.04	90
	R	0.13±0.02	0.09±0.02	18	0.20±0.03	0.16±0.04	33
Pyramis	L	0.18±0.01	0.14±0.01	25	0.19±0.03	0.13±0.03	14
	R						
Tuber	L	0.17±0.02	0.13±0.02	5	0.24±0.03	0.19±0.03	9
Uvula	L	0.17±0.01	0.13±0.01	18	0.17±0.03	0.11±0.03	30
	R						

The amplitude of BOLD signal changes of regions that belong to the 'stronger' and 'weaker' regions in Figure5 for NO PSGS and PSGS is listed.

Abbreviations: BA-Brodmann area; L-left; R-right.
